# Partial protection from fluctuating selection leads to evolution towards wider population size fluctuation and a novel mechanism of balancing selection

**DOI:** 10.1098/rspb.2023.0822

**Published:** 2023-06-28

**Authors:** Yuseob Kim

**Affiliations:** Division of EcoScience and Department of Life Science, Ewha Womans University, Ewhayeodae-gil 52, Seodaemun-gu, Seoul 03760, Korea

**Keywords:** fluctuating selection, balancing selection, storage effect, absolute fitness, population size

## Abstract

When a population is partially protected from fluctuating selection, as when a seed bank is present, variance in fitness will be reduced and reproductive success of the population will be promoted. This study further investigates the effect of such a ‘refuge’ from fluctuating selection using a mathematical model that couples demographic and evolutionary dynamics. While alleles that cause smaller fluctuations in population density should be positively selected according to classical theoretic predictions, this study finds the opposite: alleles that increase the amplitude of population size fluctuation are positively selected if population density is weakly regulated. Under strong density regulation with a constant carrying capacity, long-term maintenance of polymorphism caused by the storage effect emerges. However, if the carrying capacity of the population is oscillating, mutant alleles whose fitness fluctuates in the same direction as population size are positively selected, eventually reaching fixation or intermediate frequencies that oscillate over time. This oscillatory polymorphism, which requires fitness fluctuations that can arise with simple trade-offs in life-history traits, is a novel form of balancing selection. These results highlight the importance of allowing joint demographic and population genetic changes in models, the failure of which prevents the discovery of novel eco-evolutionary dynamics.

## 1. Introduction

Natural populations face cyclic or random fluctuations in their abiotic and biotic environments that lead to fluctuations in individuals' fitness [1–3]. Evolution under fluctuating selection has been a major topic in population genetics [4–8]. Recently, balancing selection under seasonal fluctuation of fitness has received particular attention as a potential explanation for the apparent seasonal oscillation of allele frequencies at over a thousand nucleotide sites in temperate *Drosophila melanogaster* populations [9–11]. One area of theoretical development focuses on the effect of a subset of the population or a particular life cycle stage protected from fluctuating selection that increases species or allelic diversity [12–16], which is commonly called the storage effect. Simple mathematical models showed that negative frequency-dependent selection emerges in the presence of a refuge from selection, such as a seed bank [17]. This effect can generate stable long-term polymorphism at multiple loci [18], although it requires strong selection (a large magnitude of fitness oscillation [16]). The storage effect is now broadly defined as the promotion of species or allelic diversity that arises under the non-additivity of fluctuating environment and competition on per capita growth rate and covariance between environment and competition [19–22]. Partial protection from selection is just one of many conditions for the storage effect. In this study, however, I consider only partial protection from selection and thus use the term storage effect in the narrow sense accordingly.

Partial protection from fluctuating selection is expected not only with seed banks but also in other conditions commonly encountered by plants and animals. The survival of individuals in diapause, for example in the insect pupal stage, is probably unaffected by a variable environment [23]. In species with overlapping generations, only a portion of a population will be subject to selection on a trait that is expressed at a particular stage of the life cycle [12,13]. In dioecious species, alleles affecting female reproductive traits, for example, are protected from selection if they are carried by males [24]. If an allele conferring genetic robustness or phenotypic plasticity is segregating in a population, it constitutes a genetic background on which new mutants are protected from selection [25]. In all of these cases, a species effectively consists of a subpopulation exposed to selection and another subpopulation, the refuge, protected from selection leading to the storage effect. Individuals (i.e. alleles) move between these subpopulations with a certain probability per unit time.

Temporally variable environments most likely cause a fluctuation not only in the relative fitness among alleles but also in their absolute fitness, the per capita rate of reproduction per unit time. Namely, the evolutionary change of a population under a fluctuating environment must occur while population size is changing concurrently. If population density regulation, which is defined as the force that pushes the population size towards a carrying capacity set by environmental conditions at a given time, is not very strong, population size can also change (e.g. overshoot or undershoot the carrying capacity) as its genetic composition changes. However, theoretical models for the storage effect by partial protection from selection have considered an infinitely large or constant-sized population, under which the evolutionary dynamics are fully described by the relative fitness and the relative frequencies of alleles [15,16]. This study uses a model based on absolute fitness, which allows joint changes in demography and allele frequencies. Then, it can be investigated whether and how evolutionary changes interact with fluctuations in population size. First, it remains to be examined whether the storage effect can still promote polymorphism under population size fluctuations that are allowed under relaxed density regulation. Second, one may ask whether the nature of population size fluctuation is altered by an evolutionary process. The proximate cause of population size fluctuation, observed in a wide range of species in nature, is probably the fluctuation of abiotic and biotic factors in the environment. However, the degree of such demographic fluctuation depends on the phenotypes of individuals that are the product of evolution.

Classical studies in population genetics found that the long-term reproductive success of a population or an allele under a fluctuating environment is given by the geometric mean of its fitness over time. The geometric mean decreases as fitness fluctuates in time even if its arithmetic mean remains constant. Therefore, it is predicted that a genotype causing less fluctuation in absolute fitness, for example by allocating resources for survival during a harsh period, is favoured under fluctuating environment [26,27]. It should be noted that seed banks and other means of partial protection from fluctuating selection were proposed as a means of increasing the geometric mean fitness of a population by reducing the variance of absolute fitness, a mechanism known as evolutionary bet-hedging [28,29].

Assuming that there are two alleles whose absolute fitness fluctuate differently but yield the same arithmetic or geometric mean fitness outside the refuge, I found that an allele with a wider fluctuation in absolute fitness can be positively selected under weak or moderate regulation of population density. Namely, directional, not negative frequency-dependent, selection occurs and results in the amplification of population size fluctuation. More surprisingly, pre-existing oscillation in population size was found to generate positive selection for an allele, when its relative fitness and population size oscillate in phase, leading to its fixation or long-term oscillation at intermediate frequencies. The latter is a novel mechanism of balancing selection that may explain the seasonal oscillation of allele frequencies at multiple loci observed by Bergland *et al*. [9].

The main results will be given in two steps. The full model will be first explored using numerical iterations, which reveal various patterns of eco-evolutionary dynamics. Then, theoretical explanations for these results will be sought through mathematical analyses of reduced models.

## 2. Models and Results

### (a) Modelling the fitness of a mutant allele under fluctuating selection

Consider a population of haploid individuals reproducing in discrete generations in an environment that cyclically (e.g. seasonally) changes with a period of *λ* generations. In the following, analyses will focus on the fate of a focal allele, *A*_2_, at a locus that arose recently by mutation so that its frequency, *q*, is low initially. The ancestral (wild-type) allele of the locus is *A*_1_ with frequency *p* = 1 − *q*. A trade-off is assumed in the effect of *A*_2_ on fitness over different phases of an environmental cycle. For example, due to the allocation of finite resource over time, if *A*_2_ increases fitness in one season, it inevitably leads to a decline in fitness in the other season. Here, the fitness of an *A*_2_-carrying individual can be defined relative to that of an *A*_1_-carrying individual or as the expected number of offspring per capita (the absolute fitness). In the (implicit) assumption of a strictly constant-sized population, one only needs to specify the relative fitness of alleles in the model. However, to model the joint dynamics of evolutionary and demographic changes, absolute fitness of each allele needs to be specified. Here the model of a constant (or infinite) sized population will be first considered.

First, the fitness of *A*_2_ relative to *A*_1_ in generation *t* may be given by $w_{2}=e^{s_{t}}$ (*w*_1_ = 1), where

1.1 $$s_{t}=s_{\max}\sin\left[\frac{2\pi t}{\lambda }\right].$$

This ensures that the geometric mean fitness of *A*_2_ is equal to that of *A*_1_ because the relative frequencies of *A*_1_ and *A*_2_ after one cycle of the fluctuating environment are given by *p’* and *q’*, where

$$\frac{q^{\prime }}{{\;p}^{\prime }}=\left(\overset{\lambda }{\underset{t=1}{\prod }}\frac{w_{2}}{w_{1}}\right)\frac{q}{p}=\exp\left[\overset{\lambda }{\underset{t=1}{\sum }}s_{t}\right]\frac{q}{p}=\frac{q}{p}.$$

Therefore, allele frequencies are not expected to change in this population. In this case, *A*_1_ and *A*_2_ may be called quasi-neutral to each other. There are other ways to specify the fitness of *A*_1_ and *A*_2_ so that their geometric mean fitness are identical. For example, Gulisija & Kim [15] assumed *w*_1_ = 1 − *s_t_* and *w*_2_ = 1 + *s_t_* and Bertram & Masel [16] also used an analogous model where the ratio of two alleles' fitness is simply reversed upon a seasonal change. This class of fitness assignments may be called geometric mean-preserving fitness fluctuation, or GMF in short. In the following a mutant allele having this fitness effect will be called a GMF allele.

Second, one may assume *w*_1_ = 1 and *w*_2_ = 1 + *s_t_*, making the arithmetic mean fitness of two alleles identical. Here, the fitness ratio is not reversed in the opposite phase of a cycle (e.g. 1 + *s* < 1/(1 − *s*)). In this case, it can be easily shown that *q*^′^/*p*^′^ < *q*/*p* after one environmental cycle with *s*_max_ > 0. Namely, the mutant allele *A*_2_ will be eliminated from the population since its geometric mean fitness is lower than that of *A*_1_. This fitness assignment is called arithmetic mean-preserving fitness fluctuation or AMF. A mutant allele with this fitness effect will be called an AMF allele.

Which fitness model, GMF or AMF, should be assumed is not a trivial matter as the outcomes of the following models critically depend on it. Which one is more realistic and thus should arise more often needs to be addressed as well. Under strict density regulation in a constant-sized population, *w*_2_/*w*_1_ is approximately the absolute fitness of *A*_2_ when it is rare. Then, AMF means that a fitness gain (i.e. an excess in the number of offspring) in one phase is just enough to compensate for the loss in another phase within an environmental cycle. This may happen naturally if a finite resource limits the total number of offspring born in one environmental cycle. In the case of GMF, the arithmetic mean of *w*_2_/*w*_1_ over a cycle is greater than 1 (e.g. (*e^s^* + *e*^−^*^s^*)/2 > 1 for *s* > 0). Thus, modelling with GMF means that the carrier of a rare mutant allele is assumed to produce more offspring, averaged over a cycle, than that of the wild-type allele. One may argue that such a positive effect on fitness may not be achieved by simply changing resource allocation over time. However, there can be many other ways in which fitness fluctuation arises (see more in Discussion). Why the frequency of *A*_2_ does not increase with GMF, despite increased production of offspring, was well explained by Gillespie [27] and others who showed that the outcome of fluctuating selection is determined by the geometric mean fitness, which decreases as variance in fitness fluctuation increases: *A*_2_ at low frequency has a higher mean but a larger variance in fitness compared to *A*_1_.

### (b) Storage effect under the fluctuation of relative fitness

Next, I give a brief overview of the storage effect [12,15,16]. A GMF allele, *A*_2_, is initially assumed in the fluctuating environment: $w_{2}=e^{s_{t}}$ and *w*_1_ = 1. The population is now divided into two with fractions 1-*r* and *r*. Only the first subpopulation resides in an environment subject to the fluctuating selection, which I will call the ‘field’. In the second subpopulation, individuals have identical fitness regardless of alleles and thus are protected from a fluctuating selection. While this ‘refuge’ from selection primarily models a seed bank, it may also correspond to a certain stage of a life cycle in species with overlapping generations, a spatially separated population with relaxed selection, or one sex that is not the target of sex-limited selection. Many such scenarios are true for diploid species, but the current haploid model should be applicable to diploids with incomplete dominance. It is also assumed that individuals are redistributed into the field and the refuge, with probability 1-*r* and *r*, respectively, during the migration step of each generation. Let *q_t_* be the frequency of *A*_2_ at generation *t*. It is again assumed that *A*_2_ recently arose by mutation and therefore *q_t_* is close to 0. After one cycle of fluctuating selection, frequency *q*_*t*+*λ*_ is expected to be larger than *q_t_* because

1.2 $$\begin{array}{rl} & q_{t+\lambda }=q_{t}\overset{\lambda }{\underset{i=1}{\prod }}\left((1-r)\frac{w_{2}}{\bar{w}}+r\right),\\ & \approx q_{t}\overset{\lambda }{\underset{i=1}{\prod }}\left((1-r)\frac{w_{2}}{w_{1}}+r\right)=q_{t}\exp\left[\overset{\lambda }{\underset{i=1}{\sum }}\log[(1-r)\frac{w_{2}}{w_{1}}+r]\right],\\ & \geq q_{t}\exp\left[\overset{\lambda }{\underset{i=1}{\sum }}\left((1-r)\log\frac{w_{2}}{w_{1}}+r\log1\right)\right]\\ & =q_{t}{\left(\overset{\lambda }{\underset{i=1}{\prod }}\frac{w_{2}}{w_{1}}\right)}^{1-r}=q_{t}, \end{array}$$

where multiplication/summation is over time (generation within an environmental cycle indexed by *i*). The mean fitness in the field $\bar{w}=pw_{1}+qw_{2}$ is approximated by *w*_1_ with *q* ≪ *p*. Since the logarithm is a concave function, Jensen's inequality was applied above. No change in allele frequency (*q*_*t*+*λ*_ = *q_t_*) occurs if the fluctuating selection is turned off (*s*_max_ = 0). Therefore, the presence of a refuge confers an advantage to a rare allele under fluctuating selection, thus generating the storage effect.

Furthermore, the above analysis suggests that to generate a storage effect the change of environment in the field (fluctuation of *w*_2_ versus *w*_1_) does not have to be periodic as long as the product of *w*_2_/*w*_1_ over successive generations converges to 1 with GMF. To confirm this, iteration of allele frequencies according to the model above was made using *s_t_* = *s*_max_sin[2πt*/*λ*], where t* is a random integer between 1 and *λ* drawn each generation. Interestingly, the rare allele increased in frequency slightly faster on average under this mode of random fluctuating selection than under the periodic (unscrambled) fluctuation (electronic supplementary material, figure S1).

The storage effect is intriguing because a rare allele is positively selected simply by moving between the field and refuge randomly, even though it is not favoured in either subpopulation. It is even possible that a slightly deleterious allele (i.e. having a smaller geometric mean fitness than the other allele in the field) is positively selected when it is rare [15]. A plain explanation for this phenomenon was that the loss of an allele from the field during an unfavourable period is buffered by its presence (storage) in the refuge; then, during the next period when the same allele is now favoured in the field, the refuge supplies this allele to the field. However, the buffering effect of a refuge should apply to the other (common) allele as well.

Comprehensive mathematical theories of storage effect in general settings have been developed mainly in the area of community ecology [19,21,22]. In this study, however, an alternative heuristic explanation focusing on the effect of partial protection from selection may be given by the theory of evolutionary bet-hedging [28,29]. According to the theory, a refuge such as a seed bank reduces the variance of reproductive success (of the total population) in a variable environment, thus increasing the geometric mean fitness. This effect of refuge should be larger for the carriers of the rare allele; as mentioned earlier, with GMF, the arithmetic mean of *A*_2_'s fitness is higher than that of *A*_1_ but its fluctuation larger than that of *A*_1_ prevents it from attaining higher geometric mean fitness than *A*_1_. The absolute fitness of *A*_2_ in the total population is the weighted sum of fitness in the field that is variable and fitness in the refuge that is not variable (equation (1.2)). Therefore, if the variance of fitness in the field is *V*, that in the total population is reduced to (1 − *r*)^2^*V*. The mean fitness of *A*_2_ also decreases due to the refuge (i.e. fitness advantage over *A*_1_ decreases by a factor of *r*). However, given the above result (*q_t_*_+1_ > *q_t_*), this effect of decreasing mean fitness is apparently less important compared to the decrease of variance so that the net effect is to elevate the geometric mean fitness of *A*_2_ over 1. On the other hand, the refuge has little effect on the geometric mean fitness of *A*_1_, the major allele, because its absolute fitness fluctuates negligibly. This explains how negative frequency-dependent selection for the storage effect arises due to partial protection from selection if GMF is assumed.

The storage effect does not arise for an AMF allele (*w*_1_ = 1 and *w*_2_ = 1 + *s_t_*). Again, a refuge reduces the variance of *A*_2_'s fitness and thus increases its geometric mean fitness when *A*_1_ is rare. However, the geometric mean fitness of *A*_2_ relative to *A*_1_ will approach only up to its arithmetic mean fitness relative to *A*_1_, which cannot exceed 1 under the assumption of AMF. In short, the presence of refuge reduces the fluctuation of absolute fitness, exhibited by a rare but not common allele, in the total population which converts an excess of arithmetic mean fitness, carried by a GMF but not AMF allele, into an excess of geometric mean fitness. Therefore, the proposal of storage effect as a mechanism of balancing selection in the previous studies critically depended on assuming a GMF, not AMF, allele in modelling fluctuating selection.

### (c) Cyclic fluctuation of absolute fitness: constant carrying capacity

The above model based on relative fitness and relative allele frequencies does not assume any particular population dynamics. However, a fluctuating environment is likely to generate fluctuations in population size as well if population density is not strictly regulated. Furthermore, the dynamics of allele frequency changes may depend on whether population size varies in time or not. I, therefore, expanded the above model to allow the sizes of subpopulations to change according to the absolute fitness of individuals under varying degrees of population density regulation.

Let *n*_1*f*_ and *n*_2*f*_ (*n*_1*r*_ and *n*_2*r*_) be the numbers of *A*_1_ and *A*_2_ individuals in the field (in the refuge). In the absence of density regulation, *n*_1*f*_ and *n*_2*f*_ are assumed to increase to *W*_1*f*_*n*_1*f*_ and *W*_2*f*_*n*_2*f*_ over a generation in the field. Namely, *W*_1*f*_ and *W*_2*f*_ are the absolute fitness of *A*_1_ and *A*_2_, which may change over time. Absolute fitness for both alleles in the refuge is given by *W_r_*. For density regulation, I define the carrying capacities of the field and refuge to be *K_f_* and *K_r_*, which may also change over time. In addition, it is assumed that, after steps of growth and density regulation, individuals in the field migrate to the refuge with probability *m_fr_* and those in the refuge migrate to the field with probability *m_rf_*. Then, the numbers of *A*_1_ and *A*_2_ individuals in the field and refuge in the next generation become

1.3*a* $$\begin{array}{rl} n_{1f}^{\prime } & =(1-m_{\;fr})W_{1f}n_{1f}R[W_{1f}n_{1f}+W_{2f}n_{2f},g_{f},K_{f}]\\ & \;+m_{rf}W_{r}n_{1r}R[W_{r}(n_{1r}+n_{2r}),g_{r},K_{r}], \end{array}$$

1.3*b* $$\begin{array}{rl} n_{2f}^{\prime } & =(1-m_{\;fr})W_{2f}n_{2f}R[W_{1f}n_{1f}+W_{2f}n_{2f},g_{f},K_{f}]\\ & \;+m_{rf}W_{r}n_{2r}R[W_{r}(n_{1r}+n_{2r}),g_{r},K_{r}], \end{array}$$

1.3*c* $$\begin{array}{rl} n_{1r}^{\prime } & =m_{\;fr}W_{1f}n_{1f}R[W_{1f}n_{1f}+W_{2f}n_{2f},g_{f},K_{f}]\\ & \;+(1-m_{rf})W_{r}n_{1r}R[W_{r}(n_{1r}+n_{2r}),g_{r},K_{r}], \end{array}$$

and

1.3*d* $$\begin{array}{rl} n_{2r}^{\prime } & =m_{\;fr}W_{2f}n_{2f}R[W_{1f}n_{1f}+W_{2f}n_{2f},g_{f},K_{f}]\\ & \;+(1-m_{rf})W_{r}n_{2r}R[W_{r}(n_{1r}+n_{2r}),g_{r},K_{r}], \end{array}$$

where

1.3*e* $$R[N,g,K]=\frac{1+g}{1+g(N/K)}$$

determines the strength of density regulation. If individuals carry only neutral alleles and the population size *N* is much smaller than its carrying capacity *K*, the population will grow at rate *g* (greater than or equal to 0). Therefore, a large value of *g* means strong density regulation, and the relative fitness-based model above is restored with *g* → ∞. Different strengths of regulation, *g_f_* and *g_r_*, are applied to the field and the refuge.

How a population evolves under this absolute fitness-based model was explored by the recursion of equation (1.3). Constant carrying capacity for the field (*K_f_* = 1000) and immediate redistribution of individuals over subpopulations (*m_fr_* = *m_rf_* = 0.5) was initially assumed. Four values of *g_f_* that represent the increasing strength of density regulation in the field were used (0.02, 0.2, 2 and 20). For the refuge, *g_r_* = 0 is initially assumed (as the model of a seed bank is primarily considered), which makes it meaningless to specify *K_r_*. Then, with *W_r_* = 1 and symmetric migrations, the size of the entire population in equilibrium should be 2*K_f_*.

**Figure 1.**
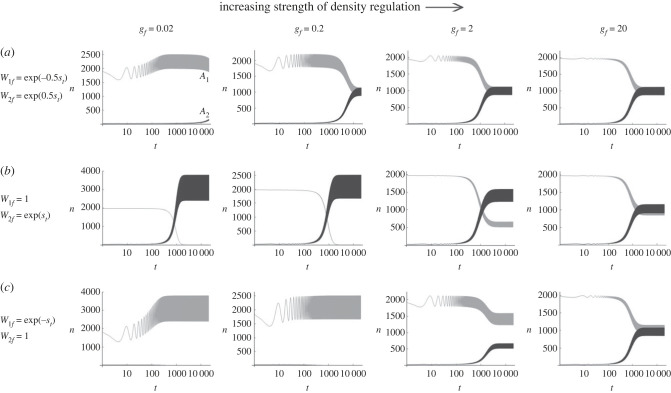
Changes in the numbers of *A*_1_ (*n*_1_ = *n*_1*f*_ + *n*_1*r*_; grey curves) and *A*_2_ alleles (*n*_2_ = *n*_2*f*_ + *n*_2*r*_; dark curves) in a population subdivided into the field and the refuge (*m_fr_* = *m_rf_* = 0.5), according to deterministic recursion of equation (1.3) starting from *n*_1*f*_ = *n*_1*r*_ = 990 and *n*_2*f*_ = *n*_2*r*_ = 10. The field maintains a constant carrying capacity (*K_f_* = 1, 000) with varying strengths (*g_f_*) of density regulation. Other parameters: *λ* = 10, *W_r_* = 1, *g_r_* = 0, *s*_max_ = 0.3.

The dynamics of a GMF allele was first explored. The fitness of *A*_1_ and *A*_2_ was given by $W_{1f}=e^{-0.5s_{t}}$, $W_{2f}=e^{0.5s_{t}}$ and *W_r_* = 1, where *s_t_* is given by equation (1.1). Namely, the oscillation in absolute fitness of the two alleles is symmetric in the field. Under this fitness scheme *n*_1_, which initially oscillated around 2*K_f_* (= 2000), decreased towards an oscillation around *K_f_* (figure 1*a*). On the other hand, *n*_2_ increased from a small to a large number that oscillates around *K_f_*. This approach of both alleles towards the relative frequency of 0.5 was faster with increasing *g*. This observation is consistent with the storage effect arising in the relative fitness-based model, which is equivalent to this absolute fitness-based model with *g* → ∞.

Next, the fitness scheme was changed to *W*_1*f*_ = 1 and $W_{2f}=e^{s_{t}}$, which keeps the relative fitness identical to the above scenario but lets the absolute fitness of *A*_2_ fluctuate more than that of *A*_1_. This dramatically changed the course of evolution (figure 1*b*). With strong density regulation (*g* = 20), the relative frequency of *A*_2_ ended up close to 0.5, as before. However, under weaker density regulation, the absolute count of *A*_2_ continued to increase towards 2*K_f_*, while that of *A*_1_ decreased towards zero. Similar results are obtained when the absolute fitness of both alleles fluctuate but the fluctuation of *A*_2_ is larger (e.g. $W_{1f}=e^{0.5s_{t}}$ and $W_{2f}=e^{1.5s_{t}}$). Namely, an allele increases in frequency by directional, not negative frequency-dependent, selection if its absolute fitness fluctuates more than that of the other allele. Switching the fitness oscillation of *A*_1_ and *A*_2_ ($W_{1f}=e^{-s_{t}}$ and *W*_2*f*_ = 1) confirmed this conclusion (figure 1*c*). Consequently, unless density regulation is strong, the population evolves towards experiencing a greater fluctuation in size.

This result—directional selection on an allele with larger fluctuation in absolute fitness—is again the consequence of assuming a GMF allele and the presence of refuge. Briefly, in the field, the arithmetic mean of $W_{2f}=e^{s_{t}}$ becomes increasingly larger than 1 as the oscillation of *s_t_* becomes larger in magnitude, while its geometric mean remains at 1. However, refuge reduces variance in the fitness of *A*_2_ in the total population, elevating its geometric mean of absolute fitness above 1.

According to a simple mathematical analysis of this model at the limit of no density regulation (*g_f_* = 0), given in the electronic supplementary material, appendix A, if the proportion of refuge is *r* in the total population and haploids are redistributed between the field and the refuge each generation (*m_fr_* = *r* and *m_rf_* = 1 − *r*), the increase in the number of an allele whose absolute fitness in the field fluctuates according to $W_{f}=e^{s_{t}}$ is given by

1.5 $$n_{T}\approx n_{0}\exp\left[\frac{1}{4}s_{\max}^{2}r(1-r)T\right],$$

where *n*_0_ is the initial number and *T* is a multiple of *λ*. This approximates well the initial increase of *A*_2_ under weak density regulation given by exact recursion (electronic supplementary material, figure S2). Equation (1.5) shows that the geometric mean fitness of a GMF allele is maximized when the size of refuge is equal to that of the field (*r* = 0.5), and that, as the rate of increase is in the order of $s_{max}^{2}$, a significant change is expected only if the amplitude of fitness fluctuation is large [16]. It is also shown in the electronic supplementary material, appendix A that this increase does not occur for mutants with AMF.

The results shown in figure 1 can now be understood in terms of the ‘intrinsic growth rate’ of a GMF allele under no density regulation described by equation (1.5). With positive (albeit weak) density regulation (*g_f_* > 0), the number of *A*_2_ cannot increase indefinitely. However, *A*_2_ outcompetes *A*_1_, whose absolute fitness fluctuates less (*W*_1*f*_ = 1 in figure 1*b*). Namely, the two alleles change in number according to their intrinsic growth rates before density regulation is applied, and their ratio determines the change in their relative frequencies. Under strong density regulation (*g_f_* ≫ 1) with constant *K_f_*, the relative frequency of *A*_2_ approaches a stable fluctuation around 0.5, at which *n*_1_ and *n*_2_ (= 2*K_f_* − *n*_1_) must fluctuate symmetrically so that their intrinsic rates of growth suggested by equation (1.5) become equal.

### (d) Cyclic fluctuation of absolute fitness: oscillating carrying capacity

**Figure 2.**
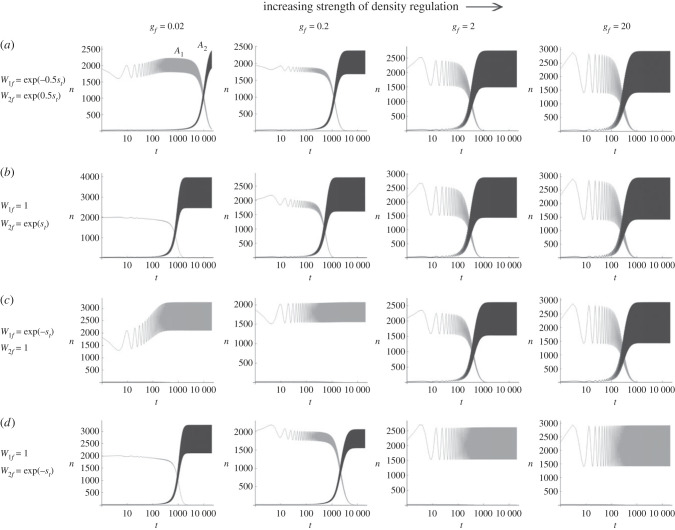
Changes in the numbers (*n*_1_ and *n*_2_) of *A*_1_ (grey curves) and *A*_2_ (dark curves) alleles under the cyclic fluctuation of carrying capacity in the field (*K_f_*_0_ = 1, 000 and *β* = 0.5). Four fitness schemes that are classified as GMF [$\left(W_{1f},W_{2f})=\;(e^{-0.5s_{t}},e^{0.5s_{t}}\right)$ (*a*), $\left(1,e^{s_{t}}\right)$ (*b*), $\left(e^{-s_{t}},1\right)$ (*c*) and $\left(1,e^{-s_{t}}\right)$ (*d*)] were used. Other parameters are identical to those in figure 1.

Next, to investigate whether pre-existing fluctuation in population size affects the evolutionary dynamics of an allele with fluctuating fitness, the carrying capacity of the field was set to change cyclically, again with period *λ*: $K_{f}=K_{\;f0}\exp[\;\beta \sin[\;2\pi t/\lambda ]]$, assuming *β*>0 without the loss of generality. This models many species' population cycles, which are commonly found to be symmetric oscillations of density in the logarithmic scale [30]. Again, a GMF allele *A*_2_ whose fitness fluctuation is in the same or opposite direction with population size fluctuation arises at *t* = 0. The fitness scheme of $W_{1f}=e^{-0.5s_{t}}$ and $W_{2f}=e^{0.5s_{t}}$, for example, means that the *A*_2_ (*A*_1_) allele acts to amplify (dampen) the oscillation of population size beyond the level specified by the ecological factor *β* under weak density regulation. The recursion of equation (1.3) under this parameter set revealed that the *A*_2_ allele is positively selected to reach fixation, surprisingly, even under strong density regulation (*g_f_* = 20) (figure 2*a*). Similar rates of increase in *n*_2_ over *n*_1_ with *g_f_* > 1 were observed with other fitness schemes: $\left(W_{1f},W_{2f})=(1,e^{s_{t}})\;\;\mathrm{or}\;(e^{-s_{t}},1\right)$ (figure 2*b,c*). On the other hand, if *A*_2_'s fitness fluctuation is out of phase (in opposite direction) with population size fluctuation (*W*_1*f*_ = 1 and $W_{2f}=e^{-s_{t}}$; figure 2*d*) positive selection for this allele under strong density regulation did not happen. Therefore, under strong density regulation, an allele is positively selected if its fluctuation in relative fitness is in phase (in the same direction) with population size (carrying capacity) fluctuation. Further exploration revealed that the fixation of *A*_2_ however requires a strong fluctuation of *K_f_* for a given magnitude of fitness fluctuation, namely *β* > *s*_max_ (electronic supplementary material, figure S3). With a relatively weaker fluctuation of population size, the long-term maintenance of polymorphism, the storage effect, was observed.

With weak density regulation (*g_f_* = 0.02 and 0.2), the allele with a larger amplitude in absolute fitness fluctuation outcompetes the other, as in the case of constant carrying capacity (figure 1*b* and 2*b*), probably because the effect of (oscillating) carrying capacity on evolutionary dynamics diminishes as density regulation weakens. It should be noted again that, unless density regulation is very strong, the fixation of *A*_2_ leads to the increased magnitude of population size fluctuation compared to that when the population was fixed for *A*_1_ (figure 2).

**Figure 3.**
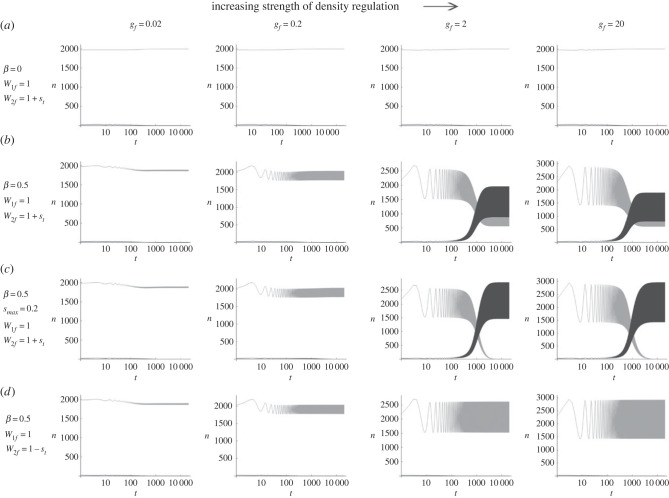
Changes in the numbers (*n*_1_ and *n*_2_) of *A*_1_ (grey curves) and *A*_2_ (dark curves) alleles when the fitness scheme of *A*_2_ is classified as AMF: (*W*_1*f*_, *W*_2*f*_) = (1, 1 + *s_t_*) (*a*–*c*), and (1, 1 − *s_t_*) (*d*). Carrying capacity in the field is either constant (*β* = 0; (*a*)) or oscillating (*β* = 0.5; (*b*–*d*)). The magnitude of fitness fluctuation, *s*_max_, is either 0.3 (*a*,*b*,*d*) or 0.2 (*c*). Other parameters are identical to those in figure 2.

Next, with AMF mutant alleles, diverse evolutionary outcomes were again obtained with oscillating carrying capacity. As confirmed earlier, *A*_2_ failed to increase in frequency when *W*_1*f*_ = 1 and *W*_2*f*_ = 1 + *s_t_* with a constant carrying capacity of the field (figure 3*a*), as its geometric mean fitness in the field alone is less than 1. However, under cyclic oscillation of *K_f_* (*β* = 0.5) and strong density regulation (*g_f_* = 2 or 20), the frequency of *A*_2_ increased by positive selection. With *s*_max_ = 0.3, *A*_2_ did not reach fixation but remained polymorphic with *A*_1_ in the long run (figure 3*b*), suggesting that a form of balancing selection arose. With a smaller *s*_max_ (= 0.2), however, *A*_2_ finally reached fixation (figure 3*c*). Again, these results depend on the condition that the fitness oscillation of *A*_2_ is in phase with population size oscillation in the field. If fitness oscillation was out of phase (*W*_1*f*_ = 1 and *W*_2*f*_ = 1 − *s_t_*), positive selection on *A*_2_ did not occur (figure 3*d*).

Finally, iterations of equation (1.3) were conducted for additional sets of scenarios. First, whether density regulation in the refuge, assumed to be absent (*g_r_* = 0) so far, has any effect on the evolutionary dynamics was examined. Imposing a strong regulation (*g_r_* = 2) keeps the size of refuge near constant while the size of field can oscillate heavily. This new condition however caused only minor changes in the dynamics observed above (electronic supplementary material, appendix B and figures S4 and S5). Second, fitness fluctuations of the alleles were given randomly while maintaining the properties of GMF or AMF in the long-term (electronic supplementary material, appendix C and figure S6). All major results observed above with cyclic fluctuation of absolute fitness were also observed with random fitness fluctuation.

### (e) Two-season model for fitness and population size fluctuation

For general understanding of positive selection generated under oscillation of carrying capacity, revealed above by numerical iterations of equation (1.3) for a limited set of parameters, a simpler model at the limit of strict density regulation is analysed. A cyclic environment with *λ* = 2 is assumed, in which each period is made up of a ‘good’ season (the first generation) and a ‘bad’ season (the second generation). The carrying capacity of the field then alternates between *K*_G_ and *K*_B_, where *K*_G_ ≥ *K*_B_. The absolute fitness of *A*_1_ and *A*_2_ individuals in the field are assumed to be *W*_1G_ and *W*_2G_ in a good season and *W*_1B_ and *W*_2B_ in a bad season. In the refuge, *W_r_* = 1. Strict density regulation in the field but no density regulation in the refuge (*g_f_* → ∞ and *g_r_* = 0) is assumed. Then, assuming *m_fr_* = *r* and *m_rf_* = 1 − *r*, this population reaches a demographic equilibrium in which the total population size alternates between *N*_G_ (at the end of a good season) and *N*_B_ (at the end of a bad season). In electronic supplementary material, appendix D, it is shown that the number of haploid individuals carrying *A*_2_ changes from *n*_2_ to $n_{2}^{\prime \prime }$ in one cycle, where

1.6 $$\begin{array}{rl} \frac{{n^{\prime \prime }}_{2}}{n_{2}} & =\left((1-r)\frac{K_{G}}{(1-r)N_{B}}\frac{W_{2G}}{W_{1G}}+r\right)\\ & \;\times \left((1-r)\frac{K_{B}}{(1-r)N_{G}}\frac{W_{2B}}{W_{1B}}+r\right), \end{array}$$

approximately, if *n*_2_ is far smaller than *n*_1_. Equation (1.6) shows that, as expected under strict density regulation, the evolutionary change of *n*_2_ is dependent on the relative, not absolute, fitness of alleles. However, because the absolute, not relative, number (*n*_2_) of individuals carrying *A*_2_ is tracked in the derivation, the growth rates of population size in the field should be combined with the relative fitness to determine its evolutionary change; the field grows from (1 − *r*)*N*_B_ to *K*_G_ during a good season and shrinks from (1 − *r*)*N*_G_ to *K*_B_ during a bad season. Here, one may define

$$\rho =\frac{K_{G}}{(1-r)N_{B}}/\frac{K_{B}}{(1-r)N_{G}}=\frac{K_{G}N_{G}}{K_{B}N_{B}},$$

as the amplitude of fluctuation in the demographic growth rate of the field.

Now, *A*_2_ is assumed to be a GMF allele. The relative fitness between two alleles is reversed each season:

$$\frac{W_{2G}}{W_{1G}}=\frac{W_{1B}}{W_{2B}}=\omega .$$

Then, the change in the number of *A*_2_ when it is rare is given by

1.7 $$f(\omega )\equiv \frac{{n^{\prime \prime }}_{2}}{n_{2}}=\left(\frac{K_{G}}{N_{B}}\omega +r\right)\left(\frac{K_{B}}{N_{G}}\frac{1}{\omega }+r\right).$$

It can be easily shown that *f*(1) = 1, as expected for a neutral allele. Solving *f*(*ω*) = 1 and analysing the derivative of *f*(*ω*) shows that

f(ω)≥1  if  r≥0andω≤1/ρ  or  ω≥1,

and *f*(*ω*) < 1 otherwise (electronic supplementary material, appendix D). Therefore, positive selection on *A*_2_ requires the presence of refuge. If *K*_G_ = *K*_B_ (no change in population size), *f*(*ω*) > 1 for all *ω* ≠ 1. Since *f*(*ω*^−1^) describes the change of *A*_1_ frequency when *A*_1_ is rare, this result means that both *A*_1_ and *A*_2_ alleles are positively selected when they are rare. This symmetry between *A*_1_ and *A*_2_ creates negative frequency-dependent selection for the storage effect. However, if *K*_G_ > *K*_B_ and 1 < *ω* < ρ, *A*_2_ is positively selected but *A*_1_ is not, even if *A*_1_ is rare. This asymmetry means that *A*_2_, whose fitness fluctuates in the same direction with population size, is under directional selection and will eventually reach fixation. For example, if *K*_G_ = 2*K*_B_ and *r* = 0.5, an allele that is between 1 and 2.5 times advantageous over the other allele during a good season but inversely disadvantageous during a bad season is positively selected until it reaches fixation. Lastly, if *ω* > ρ, both *A*_1_ and *A*_2_ alleles are positively selected when they are rare, thus yielding the storage effect again: fitness fluctuation is much stronger than population size fluctuation so that the qualitative behaviour of the system is identical to that with no population size fluctuation.

In the case of an AMF allele, we may assume (*W*_2G_/*W*_1G_) = 1 + *s* and (*W*_2B_/*W*_1B_) = 1 − *s*. The change in the number of *A*_2_ when it is rare is given by

1.8 $$\begin{array}{rl} h_{2}(s) & \equiv \frac{{n^{\prime \prime }}_{2}}{n_{2}}=\left(\frac{K_{G}}{N_{B}}(1+s)+r\right)\left(\frac{K_{B}}{N_{G}}(1-s)+r\right)\\ & =\left((1-r)\frac{k+rk}{1+rk}(1+s)+r\right)\left((1-r)\frac{1+r}{k+r}(1-s)+r\right), \end{array}$$

where *k* = (*K*_G_/*K*_B_). Solving *h*_2_(*s*) ≥ 1 gives 0 ≤ *s* ≤ (*r*/(1 − *r*^2^))(*k* − 1/*k*). Therefore, unless *s* is too large relative to the magnitude of population size fluctuation, the *A*_2_ allele whose fitness fluctuation is in phase with population growth rate invades the population. At the same time, the change in the number of *A*_1_ when it is rare is given by

1.9 $$\begin{array}{rl} h_{1}(s) & \equiv \frac{{n^{\prime \prime }}_{1}}{n_{1}}\\ & =\left((1-r)\frac{k+rk}{1+rk}\frac{1}{1+s}+r\right)\\ & \;\times \left((1-r)\frac{1+r}{k+r}\frac{1}{1-s}+r\right). \end{array}$$

**Figure 4.**
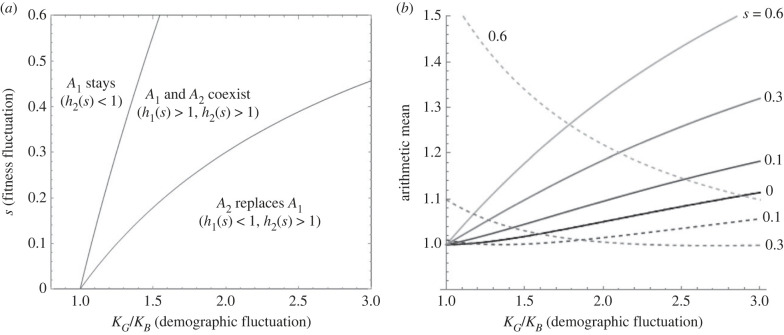
Analysis of two-season model (*r* = 0.5) with an AMF mutant. (*a*) Ranges in the amplitude of fluctuation in the carrying capacity of the field (*K*_G_/*K*_B_ = *k*) and in-phase fitness fluctuation (*s*) in which negative selection (*h*_2_(*s*) < 1), balancing selection (*h*_1_(*s*) > 1, *h*_2_(*s*) > 1) and directional selection (*h*_1_(*s*) < 1, *h*_2_(*s*) > 1) on the mutant allele *A*_2_ occurs. (*b*) The arithmetic mean of effective fitness over two seasons, of rare *A*_1_ allele (dashed curves; ((*k* + *rk*)/((1 + *rk*)(1 + *s*)) + (1 + *r*)/((*k* + *r*)(1 − *s*)))/2) and of rare *A*_2_ allele (continuous curves;((*k* + *rk*)(1 + *s*)/(1 + *rk*) + (1 + *r*)(1 − *s*)/(*k* + *r*))/2), as functions of *k* = *K*_G_/*K*_B_ with *s* = 0, 0.1, 0.3 and 0.6.

For 0 ≤ *s* ≤ *r*(*k* + 1)(*k* − 1)/((*k* + *r*)(*rk* + 1)), *h*_1_(*s*) ≤ 1 (electronic supplementary material, appendix D). Note that *r*(*k* + 1)(*k* − 1)/((*k* + *r*)(*rk* + 1)) is an increasing function of *k* ( > 1). Therefore, with *s* that is small for a given magnitude of population size fluctuation, the condition for *A*_2_ increasing in frequency to fixation is satisfied. However, with *s* > *r*(*k* + 1)(*k* − 1)/((*k* + *r*)(*rk* + 1)), *h*_1_(*s*) > 1: *A*_1_ can also invade the population. Therefore, with an intermediate strength of fluctuating selection [*r*(*k* +1)(*k* − 1)/((*k* + *r*)(*rk* + 1)) < *s* < (*r*/(1 − *r*^2^))(*k* − 1/*k*)], polymorphism with *A*_1_ and *A*_2_ should be maintained. Parameter ranges leading to different evolutionary outcomes under the assumption of AMF are graphically summarized in figure 4*a*.

Why *A*_2_ mutant allele whose absolute fitness fluctuates between 1 + *s* and 1 − *s*, which produces geometric mean fitness less than 1 so that it would be eliminated from a constant-sized population, is positively selected under in-phase oscillation in population size needs some biological explanation. Equation (1.8) suggests that the evolutionary dynamics of *A*_2_ is equivalent to that of an allele whose absolute fitness in the field fluctuates between ((*k* + *rk*)/(1 + *rk*))(1 + *s*) and ((1 + *r*)/(*k* + *r*))(1 − *s*) in a constant-sized population. One may define these as the ‘effective fitness’. Namely, demography effectively boosts/reduces the absolute fitness in the field by a factor (*k* + *rk*)/(1 + *rk*) = *K*_G_/((1 − *r*)*N*_B_) during a good season and by (1 + *r*)/(*k* + *r*) = *K*_B_/((1 − *r*)*N*_G_) during a bad season. Their ratio, ρ, quantifies how much the fitness fluctuation can be effectively amplified. (In the case of GMF mutants, the amplitude of its in-phase fitness fluctuation is simply multiplied by ρ for the effective fitness). It can be easily shown that the arithmetic mean of the effective fitness (((*k* + *rk*)(1 + *s*)/(1 + *rk*) + (1 + *r*)(1 − *s*)/(*k* + *r*))/2) is greater than 1 as long as *k* > 1 and *s* > 0 (figure 4*b* for example). Then, this arithmetic mean fitness above 1 is converted to a geometric mean fitness greater than 1 in the presence of refuge (*r* > 0), which dampens the fitness oscillation in the total population. Namely, population size fluctuation can turn an AMF mutant into an effectively GMF allele. This effect of demography modulating the effective strength of selection (the contribution of population growth rate to the absolute fitness of an allele) is analogous to that leading to modifications in the fixation probability of beneficial mutations under fluctuating population sizes [31]. Similarly, the arithmetic mean of a rare *A*_1_ allele's effective fitness (((*k* + *rk*)/((1 + *rk*)(1 + *s*)) + (1 + *r*)/((*k* + *r*)(1 − *s*)))/2; equation (1.9)) can increase to a large value if *s* approaches 1 (figure 4*b*), thus ensuring its invasion (figure 4*a*). This arithmetic mean is also above 1 when *k* is large and *s* is small. However, in this range of *s* and *k*, the arithmetic mean of *A*_2_'s fitness is much larger and its amplitude of fluctuation in the effective fitness is greater than *A*_1_ (ρ(1 + *s*)/(1 − *s*) versus ρ(1 − *s*)/(1 + *s*)). Therefore, the intrinsic growth rate of *A*_2_ should be much larger than that of *A*_1_, causing the replacement of *A*_1_ by *A*_2_.

## 3. Discussion

I modified the model of fluctuating selection in the presence of a refuge in terms of absolute fitness to accommodate the joint dynamics of population size and allele frequency changes, which is not a common practice in theoretical population genetics. However, general importance of investigating the coupled demographic and evolutionary dynamics was already well demonstrated. The outcome of natural selection under a changing population size can differ from that under a constant population size, as shown in the case of the fixation probability of beneficial alleles [31] or the nature of fitness that is maximized [32]. Conversely, various demographic changes can occur as the result of natural selection, as in the case of evolutionary rescue or trapping [33]. Furthermore, as shown in studies using simple mathematical models [34,35,36], demographic and evolutionary dynamics occur in a tightly coupled process, which is described as eco-evolutionary feedback [37,38].

This study found that the model of fluctuating selection with a refuge makes drastic changes in prediction if population size is allowed to fluctuate in response to evolutionary genetic changes. First, under weak density regulation, a GMF allele that causes a wider fluctuation in population size is under positive directional selection instead of negative frequency-dependent selection. The latter, generally defined as the storage effect, has long been proposed as a mechanism for promoting species or genetic diversity [12,16,19,22]. It was also clarified that AMF alleles are not subject to either balancing or directional selection in the presence of refuge if the carrying capacity of the population (the field) is constant over time. These results can be understood as the effect of refuge, namely partial protection from selection, that reduces the fitness fluctuation of a mutant allele in the total population, which elevates its geometric mean fitness over the wild-type. This occurs only when the arithmetic mean of mutant allele's absolute fitness in the field is already higher than that of the wild-type, which is the case of a GMF but not an AMF mutant. Greater elevation of geometric mean fitness occurs with increasing magnitude of fitness fluctuation (equation (1.5)). Then, with weak density regulation, an allele whose absolute fitness fluctuates more than the other obtains a higher intrinsic growth rate and is thus positively selected to fixation.

Positive selection for larger fluctuations in absolute fitness is consistent with a recent study [39], which used a competitive Lotka–Volterra model for investigating the dynamics of two reproductive strategies (competition between a ‘bet-hedging’ or generalist strategy that is analogous to the *A*_1_ allele in this study with smaller fitness fluctuation and a ‘rising-tide’ or specialist strategy analogous to the *A*_2_ allele with larger fitness fluctuation). Allowing population size to change according to the competitive dynamics of two strategies rather than setting it externally, they found that the rising-tide strategy is favoured under more diverse scenarios of environmental fluctuations. Their model assumed populations reproducing in overlapping generations rather than including a refuge. It should be noted that overlapping generations and partial protection from selection both reduce the variance of fitness in the total population by reducing the correlation of individuals' fitness at a given generation [40], which facilitates the conversion of a higher arithmetic mean fitness of an allele (as in the case of the rising-star strategy) into a higher geometric mean. Furthermore, other biological conditions/scenarios that reduce the fitness correlation of individuals in variable environments were found to favour variants associated with more fluctuation in fitness [41,42]. Therefore, positive selection towards larger fluctuation in population size, shown in this study to arise by partial protection from selection, is likely to occur in a broader range of biological settings.

More surprising is the result that pre-existing fluctuation in population size selects an allele, regardless of whether it is GMF or AMF, if its relative fitness fluctuates in the same direction as population size under strong density regulation. In the case of AMF mutant alleles, either negative, balancing or directional selection on them occurs depending on the magnitude of fitness fluctuation relative to population size fluctuation. To my knowledge this type of positive selection, acting on alleles that are either quasi-neutral (GMF) or deleterious (AMF) outside the refuge, induced by demographic changes has not been discovered before. The emergence of balancing selection—leading to long-term oscillation of an AMF allele at intermediate frequencies—driven by population size fluctuation is particularly remarkable. Although this mechanism also requires the presence of a refuge, it may be distinguished from the previously studied effect of partial protection from selection because the latter arises on the condition that a rare mutant allele already has higher arithmetic mean fitness than the wild-type allele, which is converted to a higher geometric mean of fitness. Here, it is the effect of fluctuating population size that elevates the ‘effective’ arithmetic mean fitness over the wild-type (figure 4*b*). Mutations to AMF alleles may arise frequently as many life-history traits are subject to trade-offs given finite resources. Then, as the whole genome is affected by population size fluctuation, positive selection on AMF alleles can arise at many loci independently as long as their fitness fluctuation is in phase with demographic fluctuation. This is a potential explanation for the seasonal oscillation of allele frequencies at more than 1000 loci in North American *Drosophila melanogaster* populations [9]. While further refinement of theory is likely needed and the ecology of these fruit flies needs to be elucidated before confirming whether this novel mechanism of balancing selection can operate in the actual population, the major requirements for the theory—clear seasonality in the temperate region and distinct stages in their life cycle, one of which can serve as a refuge—appear to be satisfied in these populations.

The fluctuation of population size in response to either a randomly or cyclically fluctuating environment is observed in many plant and animal species, particularly those reproducing in multiple generations over one seasonal cycle. Classical results in population genetics predict that a genotype causing less fluctuation in fitness is favoured under such an environment [26,27]. Namely, studies suggested that, when a mutation arises and its effect is to increase fitness in one phase of a temporally changing environment and decrease it in another phase as a trade-off, this allele is positively selected if the trade-off occurs in a direction that decreases the amplitude of population size fluctuation. Such a mutation, for example, would change the allocation of resources towards a season unfavourable for the survival and/or reproduction of a species. This study, however, showed that natural selection can occur in the opposite direction if a population is partially protected from selection. Under weak or moderate density regulation, even if the population size is initially constant over time, a GMF allele causing population size fluctuation is positively selected towards fixation. If the population size is already fluctuating due to external forces (a variable environment) and/or due to the fixation of the above size-amplifying allele at another locus, an allele causing a wider fluctuation of absolute fitness can invade and therefore amplify the fluctuation in population size under moderate density regulation (cases with *g_f_* = 0.02 and 0.2 in figures 2 and 3). Such an allele, for example, allocates further resources to a period during which the rate of growth and reproduction is already high, which inevitably causes further deterioration of fitness during other periods. Assuming a large number of loci at which mutations affecting life-history traits can arise, one can imagine that selection at multiple loci can reinforce each other until a very wide fluctuation in population size arises. For example, mast seeding, the production of a massive amount of seeds by many tree plants in certain years at irregular intervals [43,44], may be due to fixations of such alleles that maximize their reproductive output with matching resource availability.

In the theory of evolutionary bet-hedging [28,29], a refuge from selection such as a seed bank emerges in a reproductive strategy that may decrease the arithmetic mean but increase the geometric mean of fitness by reducing the variance of reproductive success. Namely, plants' delayed germination or insects' diapause can be the result of natural selection towards less severe fluctuations in fitness. In this study, however, a refuge from selection is given as a fixed demographic parameter in the model under which selection acts on genetic variants causing different patterns of fitness fluctuations. Given the current results, findings in the previous investigation on evolutionary bet-hedging may be interpreted differently. For example, by fitting the field observation of annual desert plants to their mathematical model, which closely resembles equation (1.3), Gremer & Venable [45] showed that species experiencing wider fluctuation in fitness after germination have greater proportions of their population remaining in the seed bank. This supports the hypothesis that delayed germination evolved as a bet-hedging strategy. However, it could be other life-history traits that evolved in those species; in species with a larger probability of staying in the refuge (larger seed bank), mutant alleles causing wider fluctuations in fitness in the field (after germination) could have been positively selected, as suggested in this study.

The storage effect due to partial protection from selection discovered in the previous studies and evolution towards amplified fluctuation in population size discovered in this study depend on the occurrence of mutations that cause fitness fluctuations without decreasing their geometric mean relative to the ancestral allele. Because these GMF alleles are required to produce more offspring than their ancestral alleles within an environmental cycle, they may be considered a type of beneficial allele. Such mutations probably arise less frequently than mutations generating AMF alleles, which can arise for example by simply changing resource allocation over different seasons. However, there can be many biological scenarios under which mutants' fluctuating fitness arise in a way to preserve the geometric mean. For example, imagine that body size of a haploid individual carrying *A*_2_ relative to *A*_1_ is 1 + *x* (greater than 1) in one season and 1 - *x* in the next season, as expected with the trade-off of a finite resource. Assume further that, when they encounter a predator with probability *y* for both seasons, the predator may always choose the smaller individual to attack. Then, the relative fitness of *A*_2_ becomes 1/(1 − *y*) in the first season and 1 − *y* in the second season, making *A*_2_ the GMF allele.

In conclusion, a simple mathematical model revealed novel eco-evolutionary dynamics, in which an evolutionary change leads to a demographic change (i.e. positive selection on an allele causing a wider fluctuation in population size) and a demographic change leads to an evolutionary change (i.e. population size fluctuation selecting an allele with in-phase fitness fluctuation). Such two-way interaction between ecology and evolution cannot be discovered if demography is given as a fixed parameter as in conventional population genetics models. Overall, this study extended the model of the storage effect due to partial protection from selection. Given the significant refinements and generalizations of the mathematical theory for storage effect [19,20,21,22], further investigation is warranted to interpret or reanalyse the findings of this study within the general theoretical framework.

## Data Availability

The data are provided in the electronic supplementary material [46].

## References

[1] Grant PR, Grant BR. 2002 Unpredictable evolution in a 30-year study of Darwin's finches. Science **296**, 707-711. (10.1126/science.1070315) 11976447

[2] Bell G. 2010 Fluctuating selection: the perpetual renewal of adaptation in variable environments. Phil. Trans. R Soc. Lond. B **365**, 87-97. (10.1098/rstb.2009.0150) 20008388 PMC2842698

[3] Thompson JN. 2013 Relentless evolution. Chicago, IL: University of Chicago Press.

[4] Dempster ER. 1955 Maintenance of genetic heterogeneity. Cold Spring Harbor Symposia on Quantitative Biology **20**, 25-31. (10.1101/sqb.1955.020.01.005) 13433552

[5] Haldane J, Jayakar S. 1963 Polymorphism due to selection of varying direction. J. Genet. **58**, 237-242. (10.1007/BF02986143)

[6] Hedrick PW. 1986 Genetic polymorphism in heterogeneous environments: a decade later. Annu. Rev. Ecol. Syst. **17**, 535-566. (10.1146/annurev.es.17.110186.002535)

[7] Gillespie JH. 1994 The causes of molecular evolution. Oxford, UK: Oxford University Press.

[8] Barton NH. 2000 Genetic hitchhiking. Phil. Trans. R. Soc. Lond. Ser. B **355**, 1553-1562. (10.1098/rstb.2000.0716) 11127900 PMC1692896

[9] Bergland AO, Behrman EL, O'Brien KR, Schmidt PS, Petrov DA. 2014 Genomic evidence of rapid and stable adaptive oscillations over seasonal time scales in Drosophila. PLoS Genet. **10**, e1004775. (10.1371/journal.pgen.1004775) 25375361 PMC4222749

[10] Wittmann MJ, Bergland AO, Feldman MW, Schmidt PS, Petrov DA. 2017 Seasonally fluctuating selection can maintain polymorphism at many loci via segregation lift. Proc. Natl Acad. Sci. USA **114**, E9932-E9E41. (10.1073/pnas.1702994114) 29087300 PMC5699028

[11] Machado HE et al. 2021 Broad geographic sampling reveals the shared basis and environmental correlates of seasonal adaptation in *Drosophila*. eLife **10**, e67577. (10.7554/eLife.67577) 34155971 PMC8248982

[12] Chesson PL, Warner RR. 1981 Environmental variability promotes coexistence in lottery competitive systems. Am. Nat. **117**, 923-943. (10.1086/283778)

[13] Ellner S, Hairston NG. 1994 Role of overlapping generations in maintaining genetic variation in a fluctuating environment. Am. Nat. **143**, 403-417. (10.1086/285610)

[14] Svardal H, Rueffler C, Hermisson J. 2015 A general condition for adaptive genetic polymorphism in temporally and spatially heterogeneous environments. Theor. Popul. Biol. **99**, 76-97. (10.1016/j.tpb.2014.11.002) 25446960

[15] Gulisija D, Kim Y. 2015 Emergence of long-term balanced polymorphism under cyclic selection of spatially variable magnitude. Evolution **69**, 979-992. (10.1111/evo.12630) 25707330

[16] Bertram J, Masel J. 2019 Different mechanisms drive the maintenance of polymorphism at loci subject to strong versus weak fluctuating selection. Evolution **73**, 883-896. (10.1111/evo.13719) 30883731

[17] Turelli M, Schemske DW, Bierzychudek P. 2001 Stable two-allele polymorphisms maintained by fluctuating fitnesses and seed banks: protecting the blues in *Linanthus parryae*. Evolution **55**, 1283-1298. 11525453 10.1111/j.0014-3820.2001.tb00651.x

[18] Park Y, Kim Y. 2019 Partial protection from cyclical selection generates a high level of polymorphism at multiple non-neutral sites. Evolution **73**, 1564-1577. (10.1111/evo.13792) 31273751

[19] Chesson P. 1994 Multispecies competition in variable environments. Theor. Popul. Biol. **45**, 227-276. (10.1006/tpbi.1994.1013)

[20] Schreiber SJ. 2021 Positively and negatively autocorrelated environmental fluctuations have opposing effects on species coexistence. Am. Nat. **197**, 405-414. (10.1086/713066) 33755535

[21] Johnson EC, Hastings A. 2022 Towards a heuristic understanding of the storage effect. Ecol. Lett. **25**, 2347-2358. (10.1111/ele.14112) 36181717

[22] Barabás G, D'Andrea R, Stump SM. 2018 Chesson's coexistence theory. Ecol. Monogr. **88**, 277-303. (10.1002/ecm.1302)

[23] Hedrick PW. 1995 Genetic polymorphism in a temporally varying environment: effects of delayed germination or diapause. Heredity **75**, 164-170. (10.1038/hdy.1995.119)

[24] Reinhold K. 2000 Maintenance of a genetic polymorphism by fluctuating selection on sex-limited traits. J. Evol. Biol. **13**, 1009-1014. (10.1046/j.1420-9101.2000.00229.x)

[25] Gulisija D, Kim Y, Plotkin JB. 2016 Phenotypic plasticity promotes balanced polymorphism in periodic environments by a genomic storage effect. Genetics **202**, 1437-1448. (10.1534/genetics.115.185702) 26857626 PMC4905538

[26] Lande R, Engen S, Saether BE. 2009 An evolutionary maximum principle for density-dependent population dynamics in a fluctuating environment. Phil. Trans. R. Soc. B **364**, 1511-1518. (10.1098/rstb.2009.0017) 19414466 PMC2690508

[27] Gillespie JH. 1977 Natural selection for variances in offspring numbers: a new evolutionary principle. Am. Nat. **111**, 1010-1014. (10.1086/283230)

[28] Seger J, Brockmann HJ. 1987 Oxford surveys in evolutionary biology. Oxford Surv. Evol. Biol. **4**, 182-211.

[29] Philippi T, Seger J. 1989 Hedging one's evolutionary bets, revisited. Trends Ecol. Evol. **4**, 41-44. (10.1016/0169-5347(89)90138-9) 21227310

[30] Myers JH. 1988 Can a general hypothesis explain population cycles of forest Lepidoptera? Adv. Ecol. Res. **18**, 179-242.

[31] Otto SP, Whitlock MC. 1997 The probability of fixation in populations of changing size. Genetics **146**, 723-733. (10.1093/genetics/146.2.723) 9178020 PMC1208011

[32] Sæther BE, Engen S. 2015 The concept of fitness in fluctuating environments. Trends Ecol. Evol. **30**, 273-281. (10.1016/j.tree.2015.03.007) 25843273

[33] Carlson SM, Cunningham CJ, Westley PA. 2014 Evolutionary rescue in a changing world. Trends Ecol. Evol. **29**, 521-530. (10.1016/j.tree.2014.06.005) 25038023

[34] Roughgarden J. 1971 Density-dependent natural selection. Ecology **52**, 453-468. (10.2307/1937628)

[35] Charlesworth B. 1971 Selection in density-regulated populations. Ecology **52**, 469-474. (10.2307/1937629)

[36] Lion S. 2018 Theoretical approaches in evolutionary ecology: environmental feedback as a unifying perspective. Am. Nat. **191**, 21-44. (10.1086/694865) 29244555

[37] Kokko H, López-Sepulcre A. 2007 The ecogenetic link between demography and evolution: can we bridge the gap between theory and data? Ecol. Lett. **10**, 773-782. (10.1111/j.1461-0248.2007.01086.x) 17663710

[38] Govaert L et al. 2019 Eco-evolutionary feedbacks—Theoretical models and perspectives. Funct. Ecol. **33**, 13-30. (10.1111/1365-2435.13241)

[39] Liu M, Rubenstein DR, Liu WC, Shen SF. 2019 A continuum of biological adaptations to environmental fluctuation. Proc. R. Soc. B **286**, 20191623. (10.1098/rspb.2019.1623) PMC6790776 31594502

[40] Frank SA, Slatkin M. 1990 Evolution in a variable environment. Am. Nat. **136**, 244-260. (10.1086/285094)

[41] Haaland TR, Wright J, Ratikainen II. 2019 Bet-hedging across generations can affect the evolution of variance-sensitive strategies within generations. Proc. R. Soc. B **286**, 20192070. (10.1098/rspb.2019.2070) PMC6939271 31771482

[42] Starrfelt J, Kokko H. 2012 Bet-hedging—a triple trade-off between means, variances and correlations. Biol. Rev. **87**, 742-755. (10.1111/j.1469-185X.2012.00225.x) 22404978

[43] Kelly D. 1994 The evolutionary ecology of mast seeding. Trends Ecol. Evol. **9**, 465-470. (10.1016/0169-5347(94)90310-7) 21236924

[44] Bogdziewicz M et al. 2020 From theory to experiments for testing the proximate mechanisms of mast seeding: an agenda for an experimental ecology. Ecol. Lett. **23**, 210-220. (10.1111/ele.13442) 31858712 PMC6973031

[45] Gremer JR, Venable DL. 2014 Bet hedging in desert winter annual plants: optimal germination strategies in a variable environment. Ecol. Lett. **17**, 380-387. (10.1111/ele.12241) 24393387

[46] Kim Y. 2023 Partial protection from fluctuating selection leads to evolution toward wider population size fluctuation and a novel mechanism of balancing selection. Figshare. (10.6084/m9.figshare.c.6677584) PMC10281806 37339748

